# Vitamin D deficiency in patients with cystic fibrosis: a systematic review and meta-analysis

**DOI:** 10.1186/s41043-024-00499-2

**Published:** 2024-01-17

**Authors:** Nazanin Farahbakhsh, Somaye Fatahi, Armin Shirvani, Monireh Sadat Motaharifard, Masoumeh Mohkam, Seyed Ahmad Tabatabaii, Ghamartaj khanbabaee, Shirin Yaghoobpoor, Seyedeh Zahra Davoodi, Amir hossein Hosseini

**Affiliations:** 1 Department of Pediatric Pulmonology, Mofid Pediatrics Hospital, Shahid Beheshti University of Medical Sciences, Tehran, Iran; 2 Department of Clinical Nutrition and Dietetics, Faculty of Nutrition and Food Technology, Shahid Beheshti University of Medical Sciences, Tehran, Iran; 3 Pediatric Gastroenterology, Hepatology, and Nutrition Research Center, Research Institute for Children’s Health, Shahid Beheshti University of Medical Sciences, Tehran, Iran; 4 Faculty of Medical Education, Shahid Beheshty University of Medical Sciences, Tehran, Iran; 5 Pediatric Nephrology Research Center, Research Institute for Children’s Health, Shahid Beheshti University of Medical Sciences, Tehran, Iran; 6 Student Research Committee, Faculty of Medicine, Shahid Beheshti University of Medical Sciences, Tehran, Iran

**Keywords:** Vitamin D, Immune system, CF exacerbations, Pulmonary function

## Abstract

**Aim:**

Vitamin D is a prominent modulator of immunity and respiratory function. It plays a vital role in respiratory diseases such as cystic fibrosis (CF). S. However, there is a dearth of information on patients with CF. The purpose of the meta-analysis is to highlight the importance of following the existing guidelines regarding maintenance of Vitamin D serum levels in patients with CF.

**Methods:**

The systematic search was conducted without utilizing any time or language limitations in original database from the beginning until March 2022. The meta-analysis was performed using a random-effects model. Heterogeneity was determined by *I*^2^ statistics and Cochrane *Q* test.

**Results:**

Pooled analysis using the random-effects model of the 8 case–control studies with 13 effect sizes revealed that the serum 25-OH-vitamin D in participants with cystic fibrosis was significantly lower than controls in pediatrics and adolescences (WMD: − 3.41 ng/ml, 95% CI − 5.02, − 1.80, *p* =  < 0.001) and adults (WMD: − 2.60 ng/ml, 95% CI − 4.32, − 0.89, *p* = 0.003). Based on data from 12 studies (21 effect sizes) with a total of 1622 participants, the prevalence of vitamin D levels of 20–30 ng/ml in CF patients was 36% among pediatrics/adolescents and 63% among adults. In addition, 27% of pediatric/adolescent CF patients and 35% of adult CF patients had vitamin D levels of below 20 ng/ml.

**Conclusions:**

As a result, according to the existing guidelines, our results proved the need to pay attention to the level of vitamin D in these patients.

**Supplementary Information:**

The online version contains supplementary material available at 10.1186/s41043-024-00499-2.

## Introduction

Cystic fibrosis (CF) is a disease caused by a mutation in the cystic fibrosis transmembrane conductance regulator (CFTR) gene with an autosomal recessive pattern of inheritance. This mutation impairs chloride transport across the epithelial membranes, thickening the mucus layer on the surfaces of the lung, intestines, pancreas, and other organs' surfaces [1]. This results in persistent infection and airway inflammation, leading to respiratory failure [2–4]. The exocrine and endocrine pancreas, the gastrointestinal tract, and the lungs are just a few impacted by CF. This results in lipid malabsorption and lipid-soluble vitamin deficiencies [5].

Vitamin D deficiency is common in CF patients because of fat malabsorption due to exocrine pancreatic impairment, as well as inadequate dietary intake, alterations in vitamin D metabolism, and limited sun exposure [6–9]. Despite replacing vitamin D patients who are given oral supplements, vitamin D deficiency has been reported to affect up to 90% of CF patients at [5] and the majority of patients fail to achieve therapeutic levels vitamin D deficient (< 20 ng/ml) or insufficient (20–30 ng/ml) than healthy controls.

Lower levels of circulating 25-hydroxyvitamin D [25(OH)D], a determinant of vitamin D status, are associated with a high incidence of upper respiratory tract infections and chronic lung diseases [10]. According to clinical and in vitro studies, vitamin D increases the synthesis of cathelicidin (LL–37), a critical antimicrobial peptide, showing a molecular mechanism for its immunomodulatory functions [11–13]. Numerous studies have shown that children and adults affected by CF are more likely to experience lung exacerbations when they are vitamin D deficient [18–20]. Recent pilot studies showed that vitamin D supplementation in CF patients accelerated healing following a lung exacerbation of CF [14–16]. There is no clear evidence of a direct association between vitamin D levels in children and adolescence with CF; we conducted a systematic review to assess this association.

## Methods

### Search strategy

There is a comprehensive systematic search in PubMed/MEDLINE, Web of Science, SCOPUS, and Embase from inception until March 2022 without using time or language restrictions. Keywords from the medical subject headings (Mesh) database were used for this search as follows: (vitamin D OR vit D OR ergocalciferols OR calcifediol’ OR 25-hydroxyvitamin D) AND (Cystic Fibrosis OR CF). Additionally, the reference lists of the articles retrieved, dissertation of Phd or Masters, also the gray literature, and related review studies were also hand-screened to identify eligible publications that our search might have omitted.

### Study selection

After excluding duplicate articles, two authors independently reviewed the titles, the abstracts, or the full text of the retrieved studies to detect eligible publications. Finally, original studies were included in the present meta-analysis if they met the following criteria: (1) population: adults and children of all ages and genders; (2) exposure: cystic fibrosis; (3); outcome: serum 25(OH) vitamin D; and (4) study design: cross-sectional, case–control, or cohort studies. Articles excluded from the analysis include: (1) studies not reporting the association between serum 25(OH) vitamin D and cystic fibrosis, (2) reviews, editorials, letters, or commentaries literature, (3) unpublished studies, (4) conference proceedings, (5) duplicates or irrelevant studies, and (6) animal, or in vitro or in vivo studies, and studies whose corresponding author did not offer any feedback after several emails.

### Data extraction

Two independent researchers reviewed the data and an additional reviewer resolved any disagreements between the two. The following information was collected: author, year of publication, country, age, participants’ gender, sample size, mean and standard deviation of serum 25(OH) vitamin D, prevalence of serum vitamin D deficiency and/or insufficiency.

### Quality assessment

Studies included in the meta-analysis were assessed using the Newcastle–Ottawa scale for cross-sectional and cohort studies. A quality score was specified on the basis of three major components: selection of study groups (0–5 points), adequacy of adjustment for confounding variables (0–2 points), and ascertainment of the outcome of interest (0–3 points). High-quality studies were defined as those that scored with at least seven stars on the Newcastle–Ottawa scale. Medium-quality studies received scores of five to six stars [17].

### Data synthesis and statistical analysis

The statistical analysis was conducted using STATA software version 12.0 (Stata Corp, College Station, TX, USA). Data were combined, and if there were ≥ 3 case–controls, the random-effects model was used and reported as weighted mean differences (WMDs) [18]. The prevalence of serum vitamin D deficiency and insufficiency was expressed as proportions and 95% confidence intervals (95% CI) using the random-effects model and presented as forest plot. Heterogeneity was examined using the *I*-squared (*I*^2^) statistic, in which the source of heterogeneity was determined if the *I*^2^ value was > 50%. We assessed the presence of publication bias using the formal Egger’s test [19, 20].

## Results

**Fig. 1:**
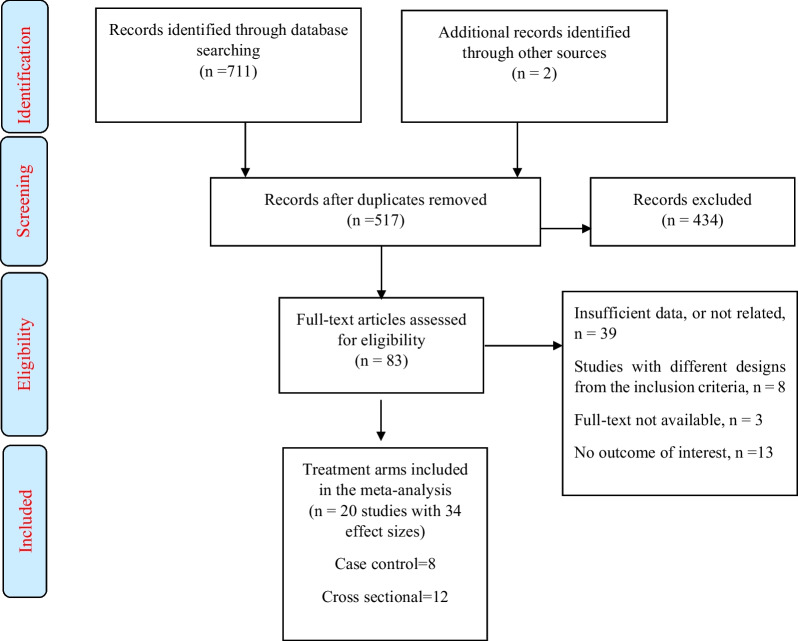
Flowchart of the selected studies, including identification, screening, eligibility and the final sample included

Figure 1 shows a flowchart of the study selection process and reasons for exclusion.. Following the systematic search, 713 publications were obtained and after excluding duplicate studies, a total of 517 publications were included. Then, we reviewed the title/abstract of the remaining studies and excluded 434 articles which did not meet the inclusion criteria. A total of 83 articles were retrieved during the secondary screening (by full text). Of these, 63 studies were discarded since they did not include data of interest. Finally, 20 studies (case–control = 8 and cross-sectional = 13) met the eligibility criteria and were included in the quantitative meta-analysis.

### Study characteristics

The characteristics of the pooled studies are presented in Tables 1 and 2. All articles were published between 1981 and 2018. Among the studies integrated in this systematic review, 8 studies were case–control [21–28] and 12 studies had a cross-sectional design [29–40]. The total sample size was 1,157 in the case control studies and 1622 patients in the cross-sectional studies. Of the 20 papers, 10 were from children, 7 from adults, and 1 included all age groups (or both children and adults). Other variables are completely presented in Tables 1 and 2.

**Table 1:** Characteristics of included cross-sectional studies

First author	Year	Country	Study population	Sex	Age	*N*	Number of individuals with vitamin D deficiency or insufficiency (%)	Number of individuals with normal vitamin D levels (%)	Deficiency or insufficiency	25OHD assay	Season adjust	Seasonality	QA
Cemlyn-Jones et al. [33]	2008	Portugal	CF	Both	26.3 ± 8	22	4 (18)	18 (81.8)	Normal range (10 to 60 ng/mL)	–	NO	Study carried out over a six-month period	7
Chirita-Emandi et al. [34]	2017	UK	CF children that had at least one DXA scan performed over a period of 10 years	Both	12.04 ± 1.89	94	37 (39)	34 (36)	Insufficiency 25–50 nmol/L	Liquid chromatography tandem mass spectrometry (LC–MS/MS) using commercial standard in human serum (Chromsystems, München, Germany)	NO	–	7
Coriati et al. [36]	2015	Canada	CF	Both	27.5 ± 8.8	178	103 (57.86)	75 (42.14)	Deficiency\insufficient (suboptimal) < 75 nmoL/L	High-performance liquid chromatography coupled with tandem mass spectrometry (TQ Detector, Waters, Milford, MA, USA)	– >	All those who had a 25(OH)D value measured at the same time were included in our study	6
Ciuna et al. [35]	2019	Romania	CF	–	–	58	43 (75)	15 (24)	25 nmol/l is generally regarded as the lower limit of normal	liquid chromatography tandem mass spectrometry	NO	–	7
Bellini et al. [32]	2021	USA	CF	Both	11.2 ± 3.6	75	17 (23)	58 (76)	Deficiency 10–30 ng/mL, insufficiency (30- 50 ng/mL	–	NO	Winter, Spring	6
Aziz et al. [31]	2021	Pakistan	CF	Both	9.45 ± 4.57	69	22 (31.88)	19 (27.53)	Deficient < 20 ng/ml	A radioimmunoassay kit	NO	All 4 seasons	7
Grey et al. [39]	2015	USA	CF	Both	12.6 ± 2.9	81	75 (95)	6 (5) < 20–40 mg/dl	25 OHD < 10 = LOW 25 OHD > 18 = Normal	–	Yes	May to October) and November to April),	7
Elkin et al. [35]	2001	UK	CF	Both	28 ± 8	104	86 (83)	18 [17]	Marginal (10.0–18.0)	–	Yes	Significantly higher values were found in the summer and autumn	6
							39 (36)	18 (17)	≤ 0 nmol/l insufficient	–	Yes	Significantly higher values were found in the summer and autumn	6
Gordon et al. [38]	2007	USA	CF	Both	31.4 ± 9.1	64	25 (39)	39 (60)	< 25 nmol/l deficiency	by radioimmunoassay (Diasorin, Stillwater, MN)	Yes	The deficiency was more common during winter: 12 cases identified with mean 25OHD for winter participants of 29.5 ± 20.2 nmol/L vs one case during summer with mean 25OHD for summer participants of 59.8 ± 23.5 nmol/L (P < 0.001)	7
Haworth et al. [41]	1999	UK	CF	Both	25.3 ± 7.1	139	53 (38)	–	< 37.5 nmol/L	–	No	All 4 seasons	7
							30 (22)	–	< 15 ng/ml insuf	–	–	–	6
							10 (7)	–	< 10 ng/ml def	–	–	–	7
Gupta et al. [40]	2017	India	CF	Both	12.8 ± 25.9	52	12 (23.1)	3 (5.77)	In patients with cystic fibrosis, total 25-OH-vitamin D levels below 30 were considered sub-optimal	25-hydroxyvitamin D (25 (OH) D) was measured using chemiluminescent immunoassay (DiaSorin LIAISON, Minnesota, USA)	NO	–	7
							37 (71.2)	3 (5.77)	15–19 ng/mL insufficient	25-hydroxyvitamin D (25 (OH) D) was measured using chemiluminescent immunoassay (DiaSorin LIAISON, Minnesota, USA)	NO	–	6
McCauley et al. [42]	2013	USA	CF	Both	8 ± 1	28	3 (11)	24 (86)	Deficiency < 30 ng/mL	Liquid chromatography/tandem mass spectrometry	NO	All 4 seasons	7
					8 ± 1	28	1 (4)	24 (86)	Sufficient (> 30 mg/L), insufficient (20–29 mg/L)	liquid chromatography/tandem mass spectrometry	NO	All 4 seasons	
					12 ± 0.75	47	15 (32)	27 (56)	Deficient (< 20 mg/L)	Liquid chromatography/tandem mass spectrometry	NO	All 4 seasons	
					12 ± 0.75	47	5 (11)	27 (56)	Sufficient (> 30 mg/L), insufficient (20–29 mg/L)	Liquid chromatography/tandem mass spectrometry	NO	All 4 seasons	
					16 ± 0.75	51	14 (27)	30 (58)	Deficient (< 20 mg/L)	Liquid chromatography/tandem mass spectrometry	NO	All 4 seasons	
					16 ± 0.75	51	7 (14)	30 (58)	Sufficient (> 30 mg/L), insufficient (20–29 mg/L)	Liquid chromatography/tandem mass spectrometry	NO	All 4 seasons

**Table 2:** Characteristics of the included case–control studies

First author	Year	Country	Study population	Sex	Multivitamins	Received vitamin D supplements	Age of cases	Age of controls	*n* case	Levels of vitamin D in cases (ng/ml) (sd)	*n* control	Levels of vitamin D in controls (ng/ml) (sd)	25OHD assay	Season adjust	Season matching	Season of measurement	QA
Ambroszkiewicz et al. [23]	2013	Poland	CF	Both	All CF children, except for 2, were pancreatic insufficient and were routinely supplemented with vitamin D3 (400 IU/day)	All or some received vitamin D supplements	7 ± 1	7 ± 1	35	19/94 ± 7.78	35	24/85 ± 8.21	Chemiluminescence immunoassay using kits from DiaSorin (USA)	NO	NR	–	6
Buntain et al. [24]	2004	Australia	CF	Both	Supplemental vitamin D in the form of a multivitamin was taken by 116 CF individuals in a mean (SD) daily dose of 4.9 (1.4) mg/day (194.7 (54.8) IU/day)	–	CHILDRENAND ADOL	Range: 5.6–48.3	95	24/3 ± 16.3	107	26 ± 16.3	DiaSorin RIA double antibody assay (DiaSorin, Stillwater, Minnesota, USA)	NO	NR	All 4 seasons	6
							ADULTS	5.6–48.3	58	21/9 ± 20	42	23/8 ± 20.49	DiaSorin RIA double antibody assay (DiaSorin, Stillwater, Minnesota, USA)	NO	NR	All 4 seasons	7
Greer et al. [25]	2003	Australia	CF	Both	–	–	8.51 ± 1.84	8.70 ± 1.50	87	25/7 ± 6.22	92	26/44 ± 6.1	DiaSorin RIA Double Antibody assay	NO	NR	–	7
							13.62 ± 2.2	13.49 ± 1.94	87	23/68 ± 6.5	92	25/7 ± 6.67	DiaSorin RIA Double Antibody assay	NO	NR	–	6
							27.00 ± 7.56	26.66 ± 7.49	62	22/11 ± 7.95	50	23/8 ± 7.54	DiaSorin RIA Double Antibody assay	NO	NR	–	6
Solomons et al. [28]	1981	USA	CF	Both	15 of 18 patients were taking supplemental vitamin D	All or some received vitamin D supplements	11 ± 3	13 ± 1.75	18	17/4 ± 8	18	23/8 ± 5.6	Competitive binding method of Haddad and Chyu (Haddad J, Chyu Ki. Competitive protein binding radioassay for 25-hydroxy-cholecalciferol. J Chin Endocrunol 197 l;33:992–5.)	YES	YES	–	6
Jakovska et al. [26]	2018	Macedonia	stable CF children	–	–	–	8.25 ± 1.9	7.5 ± 1.9	35	23/8 ± 10.9	21	25/6 ± 11.5	Electrohemiluminiscent method	NO	NR	–	7
Stead et al. [29]	1988	London	CF	Both	The mean daily intake of vitamin D was 19 1 jg (764 IU), range 0 6–54 3 pg (24–2172 IU). Only seven of the 31 patients were not taking supplements of vitamin D	All or some received vitamin D supplements	25/5	24. 5	28	10/4 ± 6.1	28	14 ± 4	Serum 25(OH)D concentration was measured by the method of Preece et al., and serum 1,25-dihydroxycholecalciferol (1,25(OH)2D) concentration by a radioreceptor assay	NO	NR	Automn	7
Thursfield et al. [30]	2018	London	CF	Both	91%(102) of CF subjects being prescribed fat-soluble vitamin supplements	All or some received vitamin D supplements	7.8 ± 4.3	12.4 ± 1.22	113	22/8 ± 15.56	6	22/8 ± 12.58	Mass spectrometry coupled with high-performance liquid chromatography	NO	NR	winter (October to March inclusive) versus summer (April to September inclusive)	7
Jakovska-Maretti et al. [27]	2013	Macedonia	stable CF patients	–	–	–	8.5 ± 2.4	Age-match controls	23	25/56 ± 12.1	70	27/75 ± 2.1	ELISA assays	NO	NR	–	7
							15 ± 2.4		23	22/07 ± 10		29/4 ± 8.3					
							25 ± 2.7		24	20/4 ± 8.4		22/78 ± 7.84					

### Meta-analysis results for case–control studies

**Fig. 2:**
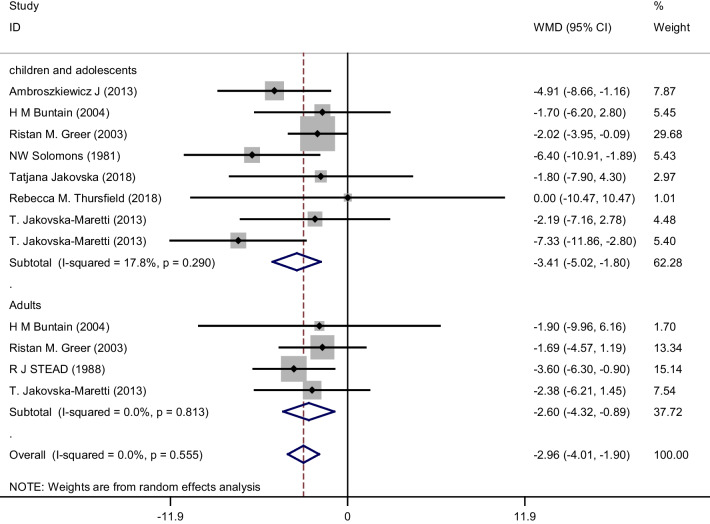
Pooled results of case–control studies for the analysis of relationship between serum 25-OH-vitamin D and cystic fibrosis

Pooled analysis using the random-effects model of the 8 case–control studies (with 13 effect sizes) revealed that the serum 25-OH-vitamin D in participants with cystic fibrosis was significantly lower than controls in pediatrics and adolescences (WMD: − 3.41 ng/ml, 95% CI − 5.02, − 1.80, *p* ≤ 0.001) and adults (WMD: − 2.60 ng/ml, 95% CI − 4.32, − 0.89, *p* = 0.003) without a significant heterogeneity seen among the papers (*I*^2^ = 17.8%, *p* = 0.29 in pediatrics and adolescences and *I*^2^ = 0%, *p* = 0.55 in adults) (Fig. 2).

### Meta-analysis results for cross-sectional studies

**Fig. 3:**
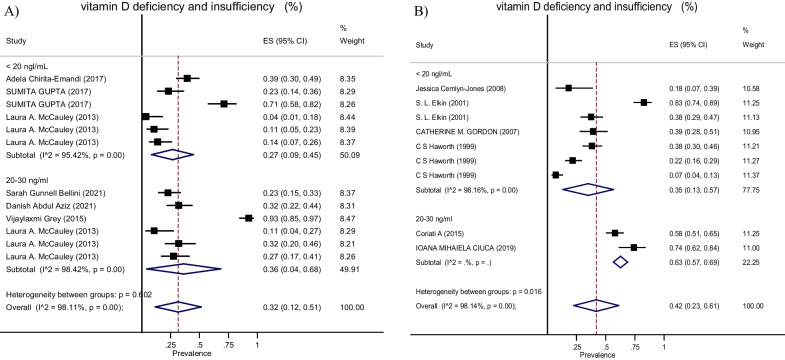
This figure shows the pooled estimate of the prevalence of vitamin D deficiency and insufficiency in **A** pediatrics and adolescents and **B** adults with cystic fibrosis patients

Based on data from 12 studies [21 effect sizes] with a total of 1622 participants, we detected an overall prevalence of vitamin D of 20–30 ng/ml in pediatrics/adolescences and adults with cystic fibrosis of 36% (95% CI 0.04, 0.68) and 63% (95% CI 0.57, 0.69), respectively, with significant heterogeneity noted among the included studies (*I*^2^ = 98.11%, *P* < 0.001; and *I*^2^ = 98.14%, *P* < 0.001; for pediatrics/adolescences and adults). The pooled prevalence of vitamin D < 20 ng/ml was 27% (95% CI 0.09, 0.45) in pediatrics/adolescences and 35% (95% CI 0.13, 0.57) in adults with cystic fibrosis (*I*^2^ = 95.42%, *P* < 0.001; and *I*^2^ = 98.16%, *P* < 0.001, respectively, for pediatrics/adolescences and adults) (Fig. 3).

### Sensitivity analysis

The leave-one-out method was applied to assess the influence of each individual study on the pooled effect size. The findings remained robust after sequential elimination of studies (Additional file 1: Fig. S1).

### Publication bias

The visual inspection of funnel plot revealed some evidence of publication bias regarding the association between serum vitamin D deficiency and insufficiency and cystic fibrosis. However, the Egger’s test also showed no evidence of bias for case–control (*P* = 0.59) or cross-sectional studies (*P* = 0.82) (Additional file 1: Fig. S2). Also, meta trim-and-fill analysis did not identify a study responsible for the publication bias (Table 3).

**Table 3:** Results of meta-analysis

	WMD	95% CI	I^2^
Pediatrics/adolescents group	− 3.41	− 5.02 to − 1.80	17.8%
Adults group	− 2.60	− 4.32 to − 0.89	0.0%

Results of cross-sectional studies meta-analysis						
	Vitamin D levels of 20–30 ng/ml			Vitamin D levels < 20 ng/m		
	Prevalence	95% CI	*I*^2^	Prevalence	95% CI	*I*^2^
Pediatrics/adolescents group	0.36	0.04 to 0.68	98.11%	0.27	0.09 to 0.45	95.42%
Adults group	0.63	0.57 to 0.69	98.14%	0.35	0.13 to 0.57	98.16%

## Discussion

It is well recognized that vitamin D deficiency and insufficiency are common in the general population and significantly more prevalent among CF patients [41]. Both the Cystic Fibrosis Foundation's and the Endocrine Society's guidelines recommend 25(OH)D levels above 30 ng/ml [8, 42]. In the current meta-analysis, CF patients had significantly lower levels of vitamin D in comparison with healthy controls in both childhood/adolescence and adulthood. We also found that the prevalence of vitamin D levels of 20–30 ng/ml in CF patients was 36% among pediatrics/adolescents and 63% among adults. In addition, 27% of pediatric/adolescent CF patients and 35% of adult CF patients had vitamin D levels of below 20 ng/ml. These results were similar to the findings of Thursfield et al. who reported that 36% of the population of children and adults had vitamin D deficiency (< 50 nmol/L).

Guidelines for treating vitamin D deficiency have been published by the Cystic Fibrosis Foundation, which recommends serum levels of 25-hydroxyvitamin D of at least 30 ng/ml. The recommendations give age-specific rising dose regimes with monitoring at 12-week intervals after switching therapies. They discuss the research on appropriate formulations (cholecalciferol rather than ergocalciferol) and vehicles of administration, as well as alternative vitamin D sources like UV lamps. Despite these comprehensive recommendations patients are still deficient [43].

Rovner et al. [5] found a 20% increase in vitamin D insufficiency in children, adolescents, and young adults with CF who were receiving regular treatment with pancreatic enzyme replacement and vitamin D supplementation. The study also used a healthy reference group whose ethnicity and latitude of residence were similar to those of the CF group and adjusted for seasonal fluctuations. Since seasonal variation in serum vitamin D levels is well recognized, comparing the degree of vitamin D insufficiency in the CF population with controls can be challenging [44]. Many studies do not standardize or take seasonal variation into account. Acute changes in 25-hydroxyvitamin D levels can also result from pancreatic enzyme compliance and vitamin D supplementation regimens employing ergocalciferol (D2) or cholecalciferol (D3).

Compared to the general population, some factors can worsen vitamin D insufficiency in people with CF. Pancreatic insufficiency causes fat malabsorption in CF patients. Absorption may be reduced even with pancreatic enzyme supplementation [45] and poor diet and noncompliance with medication. Sunlight exposure was the most accurate predictor of vitamin D deficiency before lung exacerbation, according to one study [46]; however, some individuals with CF should stay out of the sun since their use of antibiotics causes photosensitivity [47]. On the other hand, many CF patients are underweight, their bodies may not keep as much vitamin D as persons with healthy body weight [47]. Furthermore, inadequate vitamin D 25-hydroxylation in the liver and rapid enterohepatic dumping might reduce total vitamin D storage [45, 48]. Due to lower amounts of vitamin D binding protein, the primary carrier in circulation, and aid in recovering 25-hydroxyvitamin D discharged in urine, patients with CF may have impaired vitamin D storage [49]. Additionally, exposure to glucocorticoids, rifampin, and isoniazid may cause patients with CF to catabolize vitamin D more quickly [50, 51].

Clinical studies and in vitro research have provided compelling evidence that vitamin D may be crucial to the innate immune system. The antimicrobial response to bacterial toll-like receptor activation in cultured human macrophages was initially documented by Liu et al. [12]. The results of subsequent human research showed that vitamin D administration increased the local mRNA expression of cathelicidin in peripheral blood monocytes but not the levels of circulating LL-37 (a cathelicidin cleaved product) [52]. Cathelicidin mRNA expression is elevated in cultured bronchial epithelial cells with the CFTR mutation F508 in response to vitamin D therapy [53]. Furthermore, Schögler et al. [54] showed that primary bronchial epithelial cells isolated from CF patients had higher cathelicidin mRNA expression when exposed to vitamin D. They did not observe changes in the circulating protein concentrations of LL-37 in response to vitamin D in the investigation of CF patients experiencing acute pulmonary exacerbation. The use of antibiotics during pulmonary exacerbation may interfere with the vitamin D's ability to induce LL-37, which could account for the absence of alterations observed in response to vitamin D in circulation [55]. Another option is that LL-37 interacts with host-derived glycosaminoglycan, neutrophil extracellular trap DNA, and anionic bacterial compounds such endotoxin (LPS) and capsular polysaccharides [56–59]. Particularly during pulmonary exacerbations, the CF host's high quantity of bacterial-derived anionic molecules inhibits LL-37 action and may lower plasma LL-37 concentrations. Additionally, the choice to begin IV antibiotics by clinicians during an acute pulmonary exacerbation is not well standardized, which may have had an impact on their results [60]. In addition, even receiving IV antibiotics, 25–50% CF patients do not regain their baseline lung function [61, 62]. Another explanation for the absence of alterations could be that it was not possible to determine any potential local changes in lung LL-37 concentrations. It may be possible to determine whether vitamin D improves these local cellular responses by looking at the local mRNA expression of cathelicidin in peripheral blood monocytes or in monocytes and epithelial cells isolated from bronchial alveolar lavage.

VD deficiency in CF patients is associated with several complications?. Insufficient vitamin D causes associated with calcium malabsorption, resulting in secondary hyperparathyroidism. Increased parathyroid hormone levels result in calcium resorption from bone, which weakens bones, causes skeletal losses, and accelerates the onset of osteoporosis [63]. CF patients have low bone mineral density and a relatively high fracture prevalence rate of 20%, which may be related to the high rates of vitamin D insufficiency [64]. Low vitamin D levels in patients without CF are linked to a higher risk of developing cancer, autoimmune disorders, infections, and cardiovascular disease [63, 65–67].

The CF population's ability to maintain lung function may be helped by vitamin D [68]. The forced expiratory volume in one second (FEV1) and forced vital capacity (FVC), which measure lung function, showed a significant connection with vitamin D status in the Third National Health and Nutrition Examination Survey (NHANES III) [69]. In a retrospective cohort, patients with CF showed a positive correlation between vitamin D levels and FEV1 [6]. Higher vitamin D levels are associated with Hetterlung function and lower rates of pulmonary exacerbation, according to several studies on CF populations [6, 70–72]. Based on research on CF and other chronic lung diseases, vitamin D may protect lung function through enhanced airway remodeling in response to injury, lower airway inflammation, and reduced airway bacterial colonization [68].

Patients with CF are more likely to develop lung infections, frequently requiring intravenous antibiotic therapy hospitalization. The innate immune system can be strengthened by vitamin D by upregulating antimicrobial peptides like human cathelicidin (hCAP18 or its cleaved protein LL-37) [12]. Toll-like receptors on alveolar macrophages can bind to invasive bacteria, which causes an upregulation of the 1-hydroxylase and increased production of the 1,25(OH)2D and the vitamin D receptor (VDR) [73]. To eradicate the infection caused by the invasive bacteria, the locally generated 1,25(OH)2D can promote the expression of cathelicidin by macrophages and monocytes [12, 73]. In CF, locally produced 1,25(OH)2D may increase LL-37 airway concentrations, preventing bacteria like *Pseudomonas aeruginosa* and *Bordetella bronchiseptica* from colonizing the airways [53]. Additionally, vitamin D can inhibit the production of pro-inflammatory cytokines by macrophages, which may potentially lessen inflammation in the CF airway [74]. Moreover, vitamin D may have favorable effects on the development of reactive nitrogen and oxygen intermediates and the stimulation of autophagy to assist in removing infections [75].

By the time they reach adulthood, up to half of those with cystic fibrosis (CF) also have diabetes. The main cause of CF-related diabetes (CFRD) is pancreatic dysfunction, which results in inadequate insulin release and/or insulin resistance. Peng et al. found that CF adults with vitamin D deficiency are at an increased risk of developing CFRD and its earlier onset. They found that maintaining serum levels of 25(OH)D above 20 ng/mL may diminish the risk of CF progression to CFRD [76].

It is nevertheless frequent for CF patients to experience pulmonary exacerbations, which are linked to higher mortality and deterioration of lung function [77]. Previous intravenous (IV) antibiotic therapy within the last year, the length of prior IV therapy, previous hospitalization, usage of inhaled aminoglycosides, leukotriene modifiers, and high-dose ibuprofen are all factors linked to a CF pulmonary exacerbation [78]. In both adults and children with CF, nutritional issues like a lack of vitamins A, E, and D have been linked to an increased risk of pulmonary exacerbation [15, 72, 79]. Early research revealed that vitamin D may have a positive effect on lung function, inflammatory markers, and innate immunity [14–16, 54]. In previous studies, oral treatment of 250,000 IU vitamin D3 increased 1-year survival and reduced blood levels of the pro-inflammatory cytokines, (tumor necrosis factor) TNF, and IL-6, with trends toward improvements in hospital-free days and lung function (FEV1%) as compared to placebo [14, 16]. However, despite the mentioned complications, a meta-analysis by Juhasz et al. on randomized controlled trials (RCTs) showed that CF patients receiving vitamin D supplementation compared with those who did not get supplementation had no significant difference in the incidence of progressive outcomes [80].

The limitation of our results includes: the low limited number of eligible studies in our meta-analysis. We could not consider confounding factors, including ethnicity and season of vitamin D levels measurement. Another limitation was the high heterogeneity among the included cross-sectional studies.

In conclusion, our results show that CF patients have lower vitamin D levels compared to healthy controls during childhood/adolescence and adulthood. Vitamin D deficiency in these patients can potentially lead to several complications; therefore, following the guidelines on serum vitamin D levels is critical. Further research is required to determine the prevalence of vitamin D deficiency among CF patients and to improve on the current treatment regimens to maintain serum targets and prevent potential complications.

### Supplementary Information

**Additional file 1: Fig. S1.** The leave-one-out method on the pooled effect size for A) case control and , B) cross-sectional studies. **Fig. S2.** Funnel plot of the weighted mean difference (WMD) versus the standard error (s.e) for A) case control and , B)cross-sectional studies.

## Data Availability

Data will not be made available in a public repository as we have not obtained ethical clearance to share data publicly. However, on request from corresponding author data could be provided while maintaining anonymity.
