# Supplementary material for: Vitamin D deficiency in patients with cystic fibrosis: a systematic review and meta-analysis
Source: J Health Popul Nutr. 2024 Jan 17;43:11. doi: 10.1186/s41043-024-00499-2 (PMC10795301; doi:10.1186/s41043-024-00499-2)
Supplement: Supplementary file 1 — Additional file 1: Fig. S1. The leave-one-out method on the pooled effect size for A) case control and , B) cross-sectional studies. Fig. S2. Funnel plot of the weighted mean difference (WMD) versus the standard error (s.e) for A) case control and , B)cross-sectional studies. [file 41043_2024_499_MOESM1_ESM.docx]

| A)   | B)  |
| --- | --- |
| **Supplementary figure1.** The leave-one-out method on the pooled effect size for A) case control and , B) cross sectional studies. | |

| A)   | B)   |
| --- | --- |
| **Supplementary figures 2.** Funnel plot of the weighted mean difference (WMD) versus the standard error (s.e) for A) case control and , B)cross sectional studies. | |
